# Particulate Oxidative Burden as a Predictor of Exhaled Nitric Oxide in Children with Asthma

**DOI:** 10.1289/EHP175

**Published:** 2016-05-06

**Authors:** Caitlin L. Maikawa, Scott Weichenthal, Amanda J. Wheeler, Nina A. Dobbin, Audrey Smargiassi, Greg Evans, Ling Liu, Mark S. Goldberg, Krystal J. Godri Pollitt

**Affiliations:** 1 Department of Environmental Health Sciences, School of Public Health and Health Sciences, University of Massachusetts, Amherst, Massachusetts, USA; 2 Chemical Engineering and Applied Chemistry, University of Toronto, Toronto, Ontario, Canada; 3 Air Health Science Division, Health Canada, Ottawa, Ontario, Canada; 4 Department of Epidemiology, Biostatistics, and Occupational Health, McGill University, Montreal, Quebec, Canada; 5 Menzies Institute for Medical Research, University of Tasmania, Hobart, Tasmania, Australia; 6 Département de santé environnementale et de santé au travail, Université de Montréal, Montreal, Quebec, Canada; 7 Institut National de Santé Publique du Québec, Montréal, Quebec, Canada; 8 Department of Medicine, McGill University, Montreal, Quebec, Canada; 9 Division of Clinical Epidemiology, Research Institute, McGill University Health Centre, Montreal, Quebec, Canada

## Abstract

**Background:**

Epidemiological studies have provided strong evidence that fine particulate matter (PM2.5; aerodynamic diameter ≤ 2.5 μm) can exacerbate asthmatic symptoms in children. Pro-oxidant components of PM2.5 are capable of directly generating reactive oxygen species. Oxidative burden is used to describe the capacity of PM2.5 to generate reactive oxygen species in the lung.

**Objective:**

In this study we investigated the association between airway inflammation in asthmatic children and oxidative burden of PM2.5 personal exposure.

**Methods:**

Daily PM2.5 personal exposure samples (n = 249) of 62 asthmatic school-aged children in Montreal were collected over 10 consecutive days. The oxidative burden of PM2.5 samples was determined in vitro as the depletion of low-molecular-weight antioxidants (ascorbate and glutathione) from a synthetic model of the fluid lining the respiratory tract. Airway inflammation was measured daily as fractional exhaled nitric oxide (FeNO).

**Results:**

A positive association was identified between FeNO and glutathione-related oxidative burden exposure in the previous 24 hr (6.0% increase per interquartile range change in glutathione). Glutathione-related oxidative burden was further found to be positively associated with FeNO over 1-day lag and 2-day lag periods. Results further demonstrate that corticosteroid use may reduce the FeNO response to elevated glutathione-related oxidative burden exposure (no use, 15.8%; irregular use, 3.8%), whereas mold (22.1%), dust (10.6%), or fur (13.1%) allergies may increase FeNO in children with versus children without these allergies (11.5%). No association was found between PM2.5 mass or ascorbate-related oxidative burden and FeNO levels.

**Conclusions:**

Exposure to PM2.5 with elevated glutathione-related oxidative burden was associated with increased FeNO.

**Citation:**

Maikawa CL, Weichenthal S, Wheeler AJ, Dobbin NA, Smargiassi A, Evans G, Liu L, Goldberg MS, Godri Pollitt KJ. 2016. Particulate oxidative burden as a predictor of exhaled nitric oxide in children with asthma. Environ Health Perspect 124:1616–1622; http://dx.doi.org/10.1289/EHP175

## Background

The prevalence of asthma in children has increased over the past decade in developed countries (CDC 2011). Environmental factors may contribute to the development of paediatric asthma, including allergens, tobacco smoke, chemical sensitisers, diet, psychosocial factors, and indoor and outdoor air pollution (Anderson et al. 2013; Eder et al. 2006). Increased emergency department visits and hospitalizations, episodes of wheeze, as well as subclinical changes (i.e., airway inflammation) have been associated with exposure to traffic-related emissions (McCreanor et al. 2007; Zora et al. 2013), as well as specific types of particulate (ultrafine particles, black carbon) and gaseous pollutants emitted in vehicle exhaust (Cornell et al. 2012; Ostro et al. 2009). These exacerbations of asthma in children have been attributed partly to traffic-derived oxidants (McConnell et al. 2010; Sinclair et al. 2013).

Exposure to ambient particulate matter (PM) composed of redox active catalysts can induce redox-mediated oxidative stress at the air–lung interface. Multiple chemical components contribute to this oxidative response in the airway, so that assessing exposure to individual chemical constituents would be laborious for large numbers of PM samples (Li et al. 2002; Squadrito et al. 2001). Moreover, consideration of multiple compounds of a mixture in a co-pollutant model is challenged due to collinearity across species originating from common sources (Koenig et al. 2003). A measure of bulk oxidative properties as a single exposure parameter has been proposed as an integrative approach to characterizing the toxicologically relevant features of ambient PM (Ayres et al. 2008; Godri et al. 2010; Mudway et al. 2004; Weichenthal et al. 2013). This metric quantifies the oxidative burden of pollutants as their ability to oxidize constituents found in the fluid coating on the surface of the airways and the respiratory tract lining fluid. This protective network is the first point of contact that ambient pollutants have in the airway once inhaled and characterizes the capacity for the lung to tolerate radical-generating xenobiotic challenges. Antioxidants contained in this endogenous airway defense system provide a reducing environment to neutralize pollutant-induced oxidation. An acellular model of abundant low molecular antioxidants (ascorbate, reduced glutathione, and urate) was developed to replicate physiological characteristics of the respiratory tract lining fluid (Mudway et al. 2004). The capacity for ambient PM to oxidize antioxidants contained in the fluid chemical model of the respiratory tract lining is a measure of the oxidative burden of a pollutant sample.

Nitric oxide is present in the exhaled breath because it plays a number of key roles in lung biology, including as a vasodilator, bronchodilator, and inflammatory mediator (Dweik et al. 2011). Fractional exhaled nitric oxide (FeNO), which is measured noninvasively, has been validated against other markers of inflammation, including immunoglobulin E levels and blood eosinophils (Ashutosh 2000; Silvestri et al. 1999; Strunk et al. 2003), but the actual mechanisms that are indicated by FeNO are not known. It has been hypothesized that FeNO may be an indicator for up-regulation of airway inflammation (Dweik et al. 2011). FeNO is often tested in children to evaluate the presence and severity of asthma (Robroeks et al. 2007). Allergic asthmatics often exhibit elevated FeNO that increases after allergen exposures (Roos et al. 2014).

The present investigation was motivated by two studies that suggested that the respiratory health of children living in an area of Montreal, Canada, characterized by heavy industry, including two refineries, and traffic may be adversely affected (Kosatsky et al. 2004; Smargiassi et al. 2009). Rates of respiratory hospitalization among 2- to 4-year-olds were found to be 25% higher for the residential areas surrounding two oil refineries compared with the rest of Montreal. We conducted a panel study from October 2009 through April 2010, among children with asthma, 8–12 years of age, residing near these oil refineries to determine whether changes in air pollution were associated with a number of outcomes (Nethery et al. 2014; Smargiassi et al. 2014). In the present study, we determined whether daily variations in concentrations of FeNO were associated with personal exposure to fine particulate (aerodynamic diameter ≤ 2.5 μm; PM_2.5_) emissions assessed as the integrative PM oxidative burden metric.

## Methods

### Population

The target population comprised children diagnosed with asthma living in specific areas in the eastern part of Montreal near the oil refineries (Table 1). Guardians provided written, informed consent for their child to participate in the study, and the children also provided verbal assent. Ethics approval was obtained from research ethics boards at Health Canada, the McGill University Health Centre, Direction de santé publique de Montréal, and Hôpital Maisonneuve-Rosemont. Details regarding study population and recruitment have been previously described (Smargiassi et al. 2014). The present study and that by Smargiassi et al. (2014) included the same panel of children except for two additional children included in the latter; these two children were a part of a previous panel assessed in the spring of 2009.

**Table 1:** Selected characteristics of the 62 children included in the present analysis.

Variable	Value
Demographics
Median age (years) (range)	10.0 (8–12)
Sex
Boys	43 (69)
Girls	19 (31)
Race
Caucasian	40 (64.5)
Black	12 (19.4)
Other	10 (16.1)
Health status
Allergies	44 (71)
Hay fever	13 (21)
Eczema before age 2 years	17 (27)
Asthma attack in previous 12 months	31 (50)
Parental asthma	36 (58)
Medication use during monitoring
Corticosteroids	27 (44)
Rescue medication (short-acting beta agonist)	18 (29)
Median FeNO (ppb) (IQR)	16.3 (24.8)
Values are *n* (%) unless otherwise indicated.	

### Personal Pollutant Exposure Monitoring

The children carried rolling 7-kg backpacks containing personal exposure monitors and completed diaries detailing daily medication use and activities (e.g., hours spent outdoors and indoors, sports activities). Children were asked to keep the backpack with them throughout their daily activities, although no direct methods were applied to assess compliance. If they were in one location for an extended period of time (i.e., at school, playing sport, sleeping), they were instructed to keep the backpack in the same environment close to them with the sampling inlet facing up.

Personal exposure was assessed for 10 consecutive days to measure real-time concentrations of PM_2.5_ mass as well as a filter pack to sample PM_2.5_. A Hobo sensor (Hobo U10; Onset Computer Corp., Hoskin Scientific Ltd.), placed in an outside pocket of the backpack, was used to measure temperature and relative humidity.

PM_2.5_ was sampled at a flow rate of 4 LPM for 24-hr periods using a continuous PM_2.5_ monitor (pDR-1200; ThermoScientific) with an after filter (PEMs; Chempass System R&P/Thermo). We measured concentrations of PM_2.5_ from the after filter. At the daily home visit, we checked flow rates and changed batteries. If the end-flow rate of the PM_2.5_ samplers was > 20% above or below the target flow rate, corresponding continuous and gravimetric measurements were deemed invalid because both were dependent on a specific flow rate. Samples were also deemed invalid if they were deployed for > 30 hr or < 18 hr.

### Regional Pollutant Monitoring

Ambient ozone concentrations were measured hourly in Montreal at monitoring stations operated by Environment Canada through the National Air Pollution Surveillance network. Four of these stations were located in residential areas inhabited by children participating in this study.

### Characterization of Oxidative Burden

The oxidative burden of the Teflon filters was assessed. Before extraction the personal exposure filters were equilibrated for 24 hr in a weighing room with temperature (18–22°C) and humidity (45–50%) controls. Filters were weighed pre- and postextraction. The filters were submerged in high-pressure liquid chromatography grade methanol and placed in an ultrasonic bath for 15 min. Once the filters were rinsed and removed, the extractions of the samples were dried under a gentle stream of nitrogen gas in a 37°C water bath. Samples were resuspended in ultrapure water containing 5% methanol.

A synthetic human respiratory tract lining fluid, a 200 μM composite solution of physiologically relevant low-molecular-weight antioxidants (ascorbate, urate, glutathione), was incubated with PM samples for 4 hr at 37°C (Kelly et al. 2011). Positive [nonferrous dust, PD-1; National Institute of Standards and Technology (NIST), Gaithersburg, MD] and negative (model carbon black, Arosperse 15B; NIST) particle controls were assessed in parallel with the personal exposure samples to evaluate interexperimental standardisation. PM samples and controls were assessed in triplicate and incubated with the synthetic human respiratory tract lining fluid on a 96-well plate at a final concentration of 75 μg PM/mL. The extent of glutathione and ascorbate oxidation was taken to be indicative of oxidative burden, expressed as percent depletion per unit mass concentration of PM. Ascorbate oxidation was measured using absorbance spectra in an ultraviolet-visible plate reader (Molecular Devices, SpectraMax 190). Percent ascorbate depletion for each sample was measured as the change in absorbance at a wavelength of 260 nm before and after the incubation period. Following the 4-hr incubation, the reduced glutathione concentration was determined using the oxidized glutathione-reductase-5,5´-dithio-bis(2-nitrobenzoic acid) recycling assay (Baker et al. 1990; Godri et al. 2011). Glutathione-related oxidative burden was expressed as the percent difference between each sample and a 4-hr incubated particle-free blank.

### Measurement of FeNO

Single-breath, online measurements of FeNO were carried out according to the standardized procedures recommended by the American Thoracic Society and the European Respiratory Society (ATS/ERS 2005; Dweik et al. 2011) using the NIOX MINO monitor (Aerocrine) on each personal exposure day. Ambient NO was initially scrubbed with potassium permanganate by the instrument, which was then inhaled through the monitor to total lung capacity. Participants then performed a slow vital capacity maneuver by exhaling over a 6-sec period to achieve exhalation rates of 50 ± 5 mL/sec. FeNO was calculated from a minimum of two and maximum of three repeat measurement such that concentrations were within 10% or 3 ppb. We visited children’s homes each evening, approximately between 1600 and 1800 hours, to measure FeNO. Children were requested not to eat 1 hr before the FeNO measurement, and their body temperature was measured to ensure that they were not unwell.

### Statistical Methods

We used a linear mixed model, using restricted maximum likelihood estimation, to estimate the association between FeNO and personal exposures to PM_2.5_ and estimated metrics of oxidative burden over the 10-day observation period. In all models, we accounted for within-subject serial autocorrelation using a random-effect indicator for each child, a first-order autoregressive correlation structure, and indicators for day (1–10) of the study. We transformed the FeNO measurements with a natural logarithm to normalize residuals. We used the daily average value of FeNO and substituted 2.5 ppb for FeNO values below the limit of detection (5 ppb).

The effects of PM_2.5_ mass and the two oxidative burden metrics—depletion of glutathione and ascorbate—on the natural logarithm of FeNO were evaluated separately as fixed effects. The oxidative burden metrics were expressed per unit volume of air sample (% depletion/m^3^) as well as per unit PM mass (% depletion/μg). We used three exposure periods: exposure 24 hr before the FeNO measurement (the 0-day lag); exposure the day before the FeNO measurement (24–48 hr average; 1-day lag); and exposure 2 days before the FeNO measurement (48–72 hr average; 2-day lag).

We postulated that the following were potential confounding variables: personal measurements of average daily personal temperature and relative humidity, sex, corticosteroid use, use of rescue medication (short-acting beta agonists, defined as no use or any use), presence of allergies, occurrence of an asthma attack in the past year, eczema before the age of 2 years, and parental asthma. Eczema before the age of 2 years was selected as a potential confounder given the prevalence of this skin disorder in infants who go on to develop allergic asthma later in childhood (Spergel and Paller 2003). We included asthma attack in the preceding 12 months as potential covariate related to asthma severity and increased FeNO. Although the role of exposures to air pollution on the onset of eczema in infants is not clear, asthma attacks can be exacerbated in children residing in regions with elevated air pollutant concentrations (Chen et al. 2015). Corticosteroid use was defined as no reported use, irregular use, and regular use (defined as use for a minimum of 8 of the 10 study days). All covariates were considered as fixed effects. A “baseline model” that included the subject and day of study, *a priori* potential confounding factors (sex, average personal daily temperature), as well as the daily averaged exposure metrics was developed to assess the extent of confounding due to the other variables that were included in subsequent models.

We assessed possible nonlinear associations between continuous exposure variables or covariates and health outcomes using natural cubic splines with 2–5 degrees of freedom.

We added the following personal variables to the base model one variable at a time: medication use (corticosteroids, short-acting beta-agonist), presence of allergies, occurrence of an asthma attack in the first year of life, eczema before the age of 2 years, and parental asthma. Personal variables found to change the effect on the logarithm of FeNO per interquartile range (IQR) in PM_2.5_ mass or the oxidative burden metrics by ≥ 5% from the base model were included in the final model.

From the final model, we then assessed whether children’s allergies and medication use were effect modifiers with and without an interaction term; personal exposure variables were contrasted with the likelihood-ratio statistic using a maximum likelihood approach. Once an interaction was assessed to be present, restricted maximum likelihood estimation was used to estimate model parameters.

We perused diagnostics for residuals to verify that the data met the assumptions of the models, including residual autocorrelation and normality of the residuals of the random effects. The estimated associations between FeNO and the exposure metrics are expressed as the mean percent change in FeNO per increase in the IQR of the exposure metrics. No outliers were identified in the analyses.

## Results

Seventy children participated in the study, with a total of 700 possible observations. Twenty-five measurements of FeNO were missing, leaving 675 measurements. A total of 371 filters with insufficient mass loading (< 40 μg) were omitted from oxidative burden analysis, leaving 249 daily personal exposure periods for 62 children. The median number of observations per child was 3, with a range of 1–9. We compared the 62 participants included in this analysis with the entire group of 70 children (see Table S1): The median value of FeNO for the 62 children was 16.3 ppb (IQR = 24.8) when missing oxidative burden observations were removed (*n* = 249) compared with 17.7 ppb (IQR = 25.2) for the full data set. The distribution between boys and girls was also similar for the complete data set (70% of the 70 children were boys) compared with the measurements analyzed for oxidative burden (69% of the 62 children were boys).

The health status of children (presence of allergies, eczema before the age of 2 years, asthma attack in the previous 12 months, and incidence of parental asthma) included in the full and oxidative burden data sets were also comparable. On the other hand, we found that the oxidative burden data set was characterized by higher personal exposure concentrations compared with the full data set, which can be attributed to the minimum PM mass loading required for the analysis of oxidative burden.

Table 1 shows selected characteristics of the children included in the present analysis. The children were predominantly boys (69%) and Caucasian (65%). Half of the children used corticosteroids, with 24% of all subjects using corticosteroids regularly (> 8 days of the 10 day monitoring period). This medication requirement classified 15% of the study’s population with severe asthma. Of the 62 participants in this study, 41% of the children had previously been diagnosed with an allergy.

Table 2 shows details of the distribution of personal exposures to environmental variables. The median daily temperature was 21.0°C (IQR = 1.7), and the median concentration of PM_2.5_ mass across all 249 observations was 14.1 (IQR = 10.8) μg/m^3^. Day to day variations of PM_2.5_ oxidative burden were also found (ranges for ascorbate and glutathione were 0.009–0.22 and 0.0003–0.21, respectively). Ambient ozone concentrations were evaluated at four monitoring stations operated in residential regions of Montreal. Mean daily ozone concentration are presented in Figure S1. Minimal variation was found across the four residential monitoring sites.

**Table 2:** Daily personal exposure measurements of ambient particulate pollutant metrics.

Exposure	Number of samples	Mean ± SD	Median	IQR	Minimum/ maximum
Particulate matter (μg/m^3^)
PM_2.5_ mass	249	19.3 ± 16.8	14.1	10.8	6.53/101
Oxidative burden (% depletion/m^3^)
Ascorbate	249	0.08 ± 0.04	0.07	0.06	0.009/0.22
Glutathione	249	0.06 ± 0.04	0.05	0.06	0.0003/0.21
Temperature (°C)	246	21.1 ± 1.52	21.0	2.00	16.0/26.0

Ascorbate- and glutathione-related oxidative burden measures expressed per unit mass (Pearson *r* = 0.45) and per unit volume (*r* = 0.43) were correlated. No relationship was observed between PM mass concentration (μg/m^3^) and the glutathione- (*r* = –0.09) and ascorbate-related (*r* = –0.28) oxidative burden metrics (percent depletion per cubic meter). The relationship of these personal exposure metrics was further assessed with daily ambient ozone concentrations. No correlation was found with personal PM mass concentration (Pearson *r* = –0.006), glutathione-related oxidative burden (*r* = –0.1), or ascorbate-related oxidative burden (*r* = 0.06).

### Regression Analysis

Potential confounding variables were added to the baseline model (random effects for the subject and study day variables, as well as *a priori* potential confounding factors of sex, average personal daily temperature) to build the final model. Personal variables that changed the effect on FeNO by > 5% were the diagnosis of allergies, occurrence of an asthma attack in the previous 12 months, use of short-acting beta-agonists, and eczema before the age of 2 years (see Table S2A,B for the results of the models). Because these variables represent similar information, we evaluated the sensitivity of the model to inclusion of only a subset of these variables. In Table S3A,B, we present results for models adjusted by different subsets of co-variables. The addition of eczema before the age of 2 years and occurrence of an asthma attack in the previous 12 months did not yield any change in results or appreciably increase the width of the confidence intervals (CIs). They were consequently excluded from the final model. The final model was adjusted by sex, average personal daily temperature, diagnosis of allergies, and use of short-acting beta-agonists. We present in the text only the results of the final models. None of the continuous variables, including PM_2.5_ mass, ascorbate, and glutathione, were found to deviate from linearity (data not shown). We evaluated the effect of including ambient ozone concentrations in the final model for each personal exposure metric tested for the 0-day lag period (see Table S4). No difference was found in the results with the inclusion of ambient ozone. Consequently this pollutant was not included in any further analyses.

Ascorbate- and glutathione-related oxidative burden measurements were evaluated as metrics expressed per unit mass PM as well as per unit volume air sampled. Similar results were found for both mass- (see Table S2A) and volume-based metrics (see Table S2B). We present results for oxidative burden per unit volume of air sampled in the main text.

**Figure 1:**
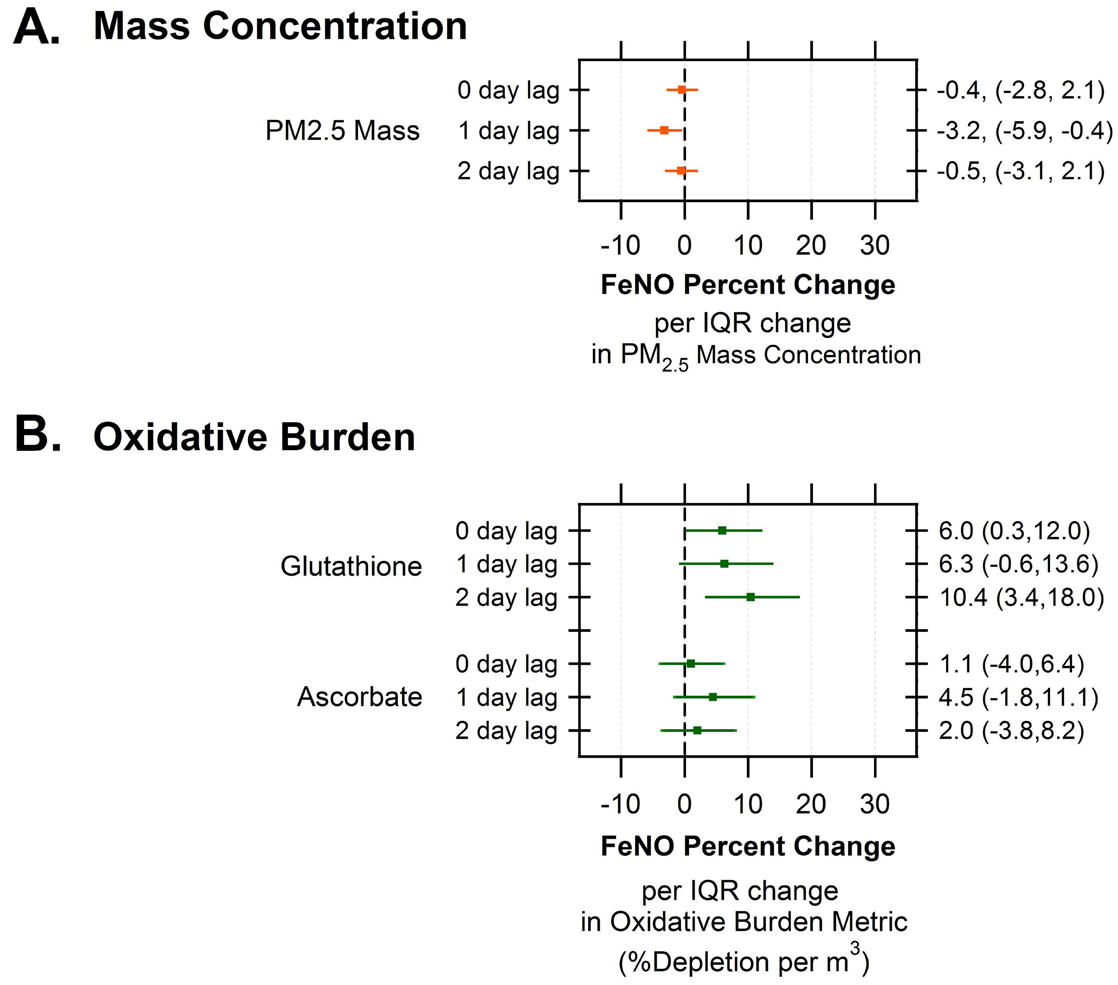
Percent change in FeNO per IQR change (95% CI) in ambient pollutant (PM_2.5_ mass concentration) (*A*) and oxidative burden (ascorbate- and glutathione-related depletion per cubic meter of air) (*B*) exposure metrics over 0-, 1-, and 2-day lags. The mixed models included a random effect for each child, a first-order autoregressive correlation structure, and indicators for day (1–10) of the study. Models were adjusted for fixed effects including temperature, sex, presence of allergies, and use of beta-agonists.

Figure 1 shows the results of the fully adjusted models for PM_2.5_ and for the two indices of oxidative stress (percent depletion per cubic meter of air), according to an increase in each variable for an increase equal to their IQRs (Table 2). For PM_2.5_, we found no associations at lags 0 and 2 days, but found a reduction in FeNO at lag 1-day (–3.2%; 95% CI: –5.9, –0.4%) per an increase of PM_2.5_ across its IQR (11.3 μg/m^3^). Across all three lags, only glutathione-related oxidative burden was found to be positively associated with FeNO: For example, at lag 0-days (past 24 hr), we found a 6.0% (95% CI: 0.3, 12.0%) increase in FeNO per IQR increase of glutathione-related oxidative burden (IQR = 0.32 depletion per m^3^).

**Figure 2:**
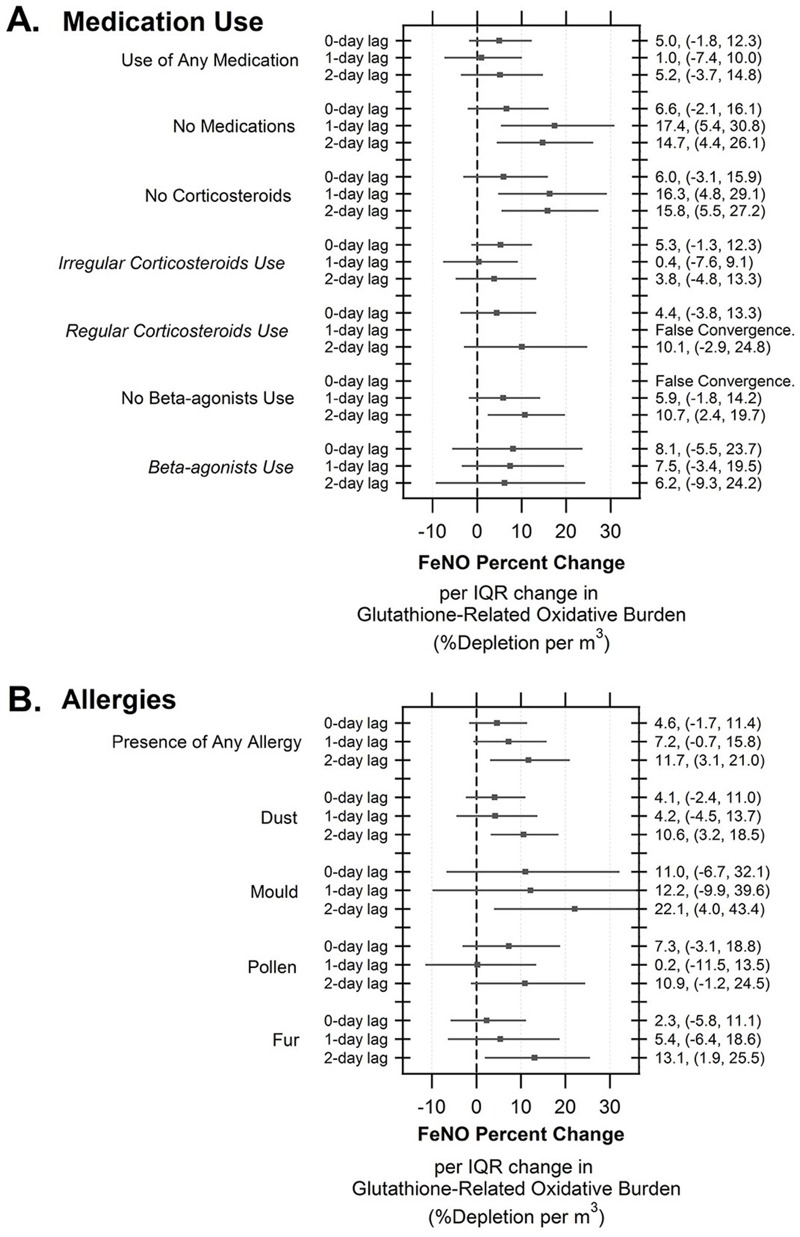
Effects of glutathione-related oxidative burden exposure per cubic meter of air on the percent change in FeNO over 0-, 1-, and 2-day lags as modified by medication use (any medication, none, corticosteroids, beta-agonists) (*A*) and presence of allergies (dust, mold, pollen, fur) (*B*). The mixed models included a random effect for each child, a first-order autoregressive correlation structure, and indicators for day (1–10) of the study. Models were adjusted for fixed effects including temperature, sex, presence of allergies and use of beta-agonists.

Figure 2 shows the results of analysis for glutathione-related oxidative burden associations according to use of asthma medications and presence of allergies, evaluated at all three lags. Children who did not use any medications experienced increased FeNO per IQR increase of glutathione for the 1-day lag (17.4%; 95% CI: 5.4, 30.8) and 2-day lag exposure periods (14.7%; 95% CI: 4.4, 26.1) as compared to children who used medications (Figure 2A; see also Table S5B), although the confidence intervals between groups overlapped (interaction 1-day lag *p* = 0.08; 2-day lag *p* = 0.21). As well, FeNO was elevated for children who did not take corticosteroids (lag-2 days: 15.8%; 95% CI: 5.5, 27.2) compared with children who used these medications irregularly (lag-2 days: 3.8%; 95% CI: –4.8, 13.3; interaction *p* = 0.11). No important differences were found for the beta-agonists. Similar results were found for glutathione-related oxidative burden measurements expressed per unit mass (see Table S5A) and per unit volume (see Table S5B). There was no evidence of effects of PM_2.5_ ascorbate-related oxidative burden or mass concentration on FeNO that were found to be modified by medication use (see Table S5B).

A suggestion of an enhanced FeNO response to glutathione-related oxidative burden exposure was found at the 2-day lag in children with allergies to dust (10.6%; 95% CI: 3.2, 18.5%), mold (22.1%; 95% CI: 4.0, 43.4%), or fur (13.1%; 95% CI: 1.9, 25.5%) (Figure 2B; see also Table S6B); however, compared with children without these allergies, no difference was found (interaction dust *p* = 0.96; mold *p* = 0.18; fur *p* = 0.36). No increase in FeNO response was found for the 0- or 1-day lag period. Similar results were found for the glutathione-related oxidative burden metric expressed per unit mass (see Table S6A). We did not observe any associations of PM_2.5_ mass or the ascorbate-related oxidative burden metrics with FeNO according to the presence of allergy (see Table S6B).

## Discussion

In this panel study of asthmatic children living in Montreal, Canada, we evaluated daily measurements of FeNO, a possible subclinical biomarker of airway inflammation, in relation to personal exposure to PM_2.5_ mass and to two metrics of oxidative stress. We found FeNO to be positively associated with a metric of oxidative burden (glutathione) but not with PM mass in asthmatic children.

The novel aspect of this study is the daily longitudinal personal monitoring of a sensitive cohort to characterize pro-oxidant pollutant exposure and its effect on FeNO. The study population comprised children residing in neighborhoods with exposures to industrial emissions and traffic sources. Other studies, discussed below, have relied on exposure data estimated for traffic-related emissions (mostly nitrogen dioxide) from land use regression models of annual averages or these studies relied on central monitoring sites.

Increases in FeNO may indicate an increase in the underlying eosinophilic inflammation that is the hallmark of asthma, before changes in clinical symptoms are observed, but the pathways are not well understood (Dweik et al. 2011). In fact, small changes in FeNO are not strongly correlated with clinical symptoms (Silvestri et al. 1999; Strunk et al. 2003), although FeNO rises dramatically during episodes of asthma exacerbation and decreases when they resolve (Ashutosh 2000). For example, Massaro et al. (1995) found that FeNO levels during an acute asthma attack requiring emergency care were about 50% higher than stable baseline levels. The above considerations must be kept in mind when interpreting the results of the present study.

We found an inverse association between PM_2.5_ mass and percent change in FeNO at the 1-day lag, but little evidence of associations at 0- or 2-day lags. It is possible that the association at lag-1 day was attributable to chance because it is especially difficult to appreciate how this environmental exposure could lead to improvements in respiratory health. There have been few panel studies investigating the effects of fine particulates on FeNO. In particular, American and Mexican asthmatic children living in proximity to the border were also reported to experience a small increase in FeNO with elevated 3-day averaged PM_2.5_ mass and nitrogen dioxide levels measured in school playgrounds (Sarnat et al. 2012). Positive associations between FeNO, collected daily over 10 consecutive days, and PM_2.5_ mass concentrations were additionally reported for a panel study of children living in Seattle, Washington (6–13 years of age) (Koenig et al. 2003). Increased FeNO was observed for 1-day averaged PM_2.5_ mass concentration measured outside and inside the children’s home as well as using personal monitors and central monitoring sites. Further evaluation of this Seattle panel identified positive associations between 1-hr averaged PM_2.5_ mass concentrations collected from the central monitoring sites for up to 12 hr after exposure (Mar et al. 2005).

The only other studies assessing FeNO in asthmatic children that we are aware of were designed using cross-sectional comparisons. Altuğ et al. (2014) reported no association between FeNO in Dutch asthmatic children and weekly average nitrogen dioxide or sulfur dioxide exposure concentrations measured in school playgrounds (Altuğ et al. 2014). Another study conducted in Windsor, Canada, showed positive associations between FeNO and annual PM_2.5_ mass concentrations estimated at the children’s home address using a land use regression model (Dales et al. 2008).

The PM_2.5_ oxidative burden related to glutathione depletion was identified across all three lag periods to be associated with elevated FeNO in asthmatic children. Ascorbate-related oxidative burden was not found to be associated with this airway response. In a cohort of California children, Delfino et al. (2013) also reported an association between PM_2.5_ oxidative burden and FeNO measured at a central monitoring site, but no association was found between FeNO and PM_2.5_ mass. These authors found increases in FeNO of 8.7–9.9% per IQR increase of oxidative burden for 1 day and 2 day lags. Positive associations between FeNO and water soluble organic carbon based on a 2-day moving average were further described.

There are few studies in asthmatic children of oxidative burden. Delfino et al. (2013) described the only other study in asthmatic children that considered oxidative burden as an alternative exposure metric to PM mass concentration. Although similar positive associations were identified in both this California study and ours, different methods were used to quantify PM redox activity. Both methods evaluated the capacity of redox-active PM species to catalyze the reduction of oxygen to form superoxide and reactive oxygen species via subsequent reactions. We assessed the formation of these reactive oxygen species by quantifying the induced antioxidant depletion. In contrast, Delfino et al. (2013) measured the depletion of dithiothreitol. Dithiothreitol depletion has been primarily associated with polycyclic aromatic hydrocarbons and quinones (O’Brien 1991), but high concentration of metals can also oxidize dithiothreitol (Charrier and Anastasio 2012; Kachur et al. 1997). Glutathione depletion from the synthetic respiratory tract lining fluid model similarly exhibits enhanced sensitivity to organic compounds as well as redox-active metals at elevated concentrations (see Figure S2).

We observed enhanced FeNO with exposure to PM_2.5_ characterized by increased glutathione-related oxidative burden for children with allergies to mold, fur, or dust. Suggestions of enhanced exacerbations of asthmatic symptoms in children exposed to traffic-related pollutants and allergens have been reported previously for exposures to nitrogen dioxide (Dell et al. 2014), black carbon (Cornell et al. 2012), formaldehyde and acetaldehyde (Flamant-Hulin et al. 2009), as well as trace metals (nickel from residential oil heating) (Rosa et al. 2014). Ours is the first report to show that the effects of pro-oxidant exposure, quantified by oxidative burden, may be modified by the presence of allergic disease, although we must emphasize that there was no clear signal in these interaction analyses. If these findings can be replicated, they may suggest that allergic sensitization may contribute to the pathogenesis of air pollutant–induced asthma exacerbations.

Children who regularly used corticosteroids had a mean FeNO concentration of 15.1 ppb, whereas no corticosteroid use resulted in concentrations of 23.8 ppb for the asthmatic children in this study. When stratified by medication use, children not using any medications experienced the greatest increase in FeNO per IQR increase of PM_2.5_ oxidative burden. Again, we emphasize that these results are only suggestive that medication use (corticosteroids) may reduce the FeNO response induced by the pro-oxidants of PM. Increased FeNO in children not taking asthma medications and reduced inflammatory response in regular corticosteroid users have been previously reported (Mar et al. 2005).

We did not observe associations between PM_2.5_ glutathione-related oxidative burden and mass concentration, in keeping with previous exposure studies (Künzli et al. 2006; Szigeti et al. 2014). This lack of relationship suggests that certain chemical constituents of PM_2.5_ may be responsible for oxidative burden, and not bulk PM mass concentration. The pro-oxidant fraction of PM is likely attributable to the observed antioxidant depletion (Kelly et al. 2011; Shi et al. 2003).

A primary strength of this study was the detailed and repeated follow-up of a panel of participants. The same technicians visited the home each day to ensure consistency across health measurements. The 10 consecutive days of data collection enabled detailed analysis of short-term effects. Another important strength of the study was the taking of personal measurements of air pollutants and other environmental factors.

This multi-day monitoring period, however, presented challenges. In particular, daily visits placed a high demand on study participants and limited recruitment. Further challenges included low PM mass loading on personal exposure filters. A number of filter samples were excluded from the oxidative burden analysis due to insufficient PM mass collection. The missing oxidative burden data, an acknowledged caveat of our analysis, decreased power and may have also presented a bias toward children with higher pollutant exposure levels. Although we were able to observe effects on FeNO, recruitment and oxidative burden measurement limitations yielded a relatively low sample size, especially in stratified analyses examining the modifying effect of medication use and presence of allergies.

A limitation of this study is the exclusive use of the synthetic respiratory tract lining fluid method to assess oxidative burden. Other acellular methods include quantification of dithiothreitol depletion by PM-induced reactive oxygen species generation as well as measurement of hydroxyl radical generation by PM redox-active species using the electron paramagnetic resonance assay (Ayres et al. 2008). Janssen et al. (2014) considered the equivalence of oxidative burden measurements evaluated as dithiothreitol depletion, ascorbate depletion, and electron paramagnetic resonance. Univariate analysis suggested the strongest correlation between PM_2.5_ ascorbate-related oxidative burden and electron paramagnetic resonance (Spearman *r* = 0.89) (Janssen et al. 2014). Weaker correlations were reported for dithiothreitol-related oxidative burden with ascorbate-related oxidative burden (*r* = 0.63) and electron paramagnetic resonance (*r* = 0.52). The electron paramagnetic resonance method was also contrasted against antioxidant depletion by Künzli et al. (2006): A stronger correlation was found between oxidative burden measured as hydroxyl radical generation measured by electron paramagnetic resonance and ascorbate depletion (Pearson *r* = 0.65) compared with glutathione depletion (*r* = 0.18) (Künzli et al. 2006). The lack of equivalence across measurement techniques may be attributed to the sensitivity of each method to different PM chemical characteristics (Ayres et al. 2008): Glutathione and dithiothreitol depletion are most sensitive to organic species, whereas the electron paramagnetic resonance measure is primarily mediated by redox-active metals (Shi et al. 2003). The variance in sensitivity of each oxidative burden method across different organic and metal PM species must be acknowledged when interpreting reported responses.

## Conclusions

The oxidative burden metric provided a bulk measure of the pro-oxidant content of PM, and we found that one metric of PM_2.5_ oxidative burden was associated with FeNO, a biomarker suggested to be indicative of eosinophilic airway inflammation. As well, there was a suggestion that using corticosteroids may reduce the response of FeNO to exposures to oxidative burden, and some allergies may increase this airway response. We did not find any association between FeNO and personal exposure measures of PM_2.5_ mass.

## Supplemental Material

(228 KB) PDF
