# Supplementary material for: Particulate Oxidative Burden as a Predictor of Exhaled Nitric Oxide in Children with Asthma
Source: Environ Health Perspect. 2016 May 6;124(10):1616–22. doi: 10.1289/EHP175 (PMC5047770; doi:10.1289/EHP175)
Supplement: (228 KB) PDF [file EHP175.s001.acco.pdf]

Environ Health Perspect
DOI: 10.1289/EHP175

Note to readers with disabilities: *EHP* strives to ensure that all journal content is accessible to all readers. However, some figures and Supplemental Material published in *EHP* articles may not conform to 508 standards due to the complexity of the information being presented. If you need assistance accessing journal content, please contact ehp508@niehs.nih.gov. Our staff will work with you to assess and meet your accessibility needs within 3 working days.

Supplemental Material

Particulate Oxidative Burden as a Predictor of Exhaled Nitric Oxide in Children with Asthma

Caitlin L. Maikawa, Scott Weichenthal, Amanda J. Wheeler, Nina A. Dobbin, Audrey Smargiassi, Greg Evans, Ling Liu, Mark S. Goldberg, and Krystal J. Godri Pollitt

Table of Contents

Table S1. Comparison of select characteristics of the 70 children in the full data set and the 62 children included in the oxidative burden data set.

Table S2A. Percent change in FeNO per IQR for pollutant exposure metrics expressed per unit mass for a 0-, 1- and 2-day lag periods with the addition of potential confounding variables to the base, linear mixed model. The base model included fixed effects for sex and ambient temperature as well as random subject and day of study factors. The final model included additional personal variables: the presence of allergies and use of beta-agonists.

Table S2B. Percent change in FeNO per IQR for pollutant exposure metrics expressed per unit volume for a 0-, 1- and 2-day lag periods with the addition of potential confounding variables to the base, linear mixed model. The base model included fixed effects for sex and ambient temperature as well as random subject and day of study factors. The final model included additional personal variables: the presence of allergies and use of beta-agonists.

Table S3A. Comparison of the percent change in FeNO per IQR for pollutant exposure metrics expressed per unit mass for a 0-, 1- and 2-day lag periods in model using varying levels of model adjustment. The base model included fixed effects for sex and ambient temperature as well as

random subject and day of study factors. Additional personal variables were included adjusted models. The final model presented in the manuscript corresponds to Adjusted Model 1.

Table S3B. Comparison of the percent change in FeNO per IQR for pollutant exposure metrics expressed per unit volume for a 0-, 1- and 2-day lag periods in model using varying levels of model adjustment. The base model included fixed effects for sex and ambient temperature as well as random subject and day of study factors. Additional personal variables were included adjusted models. The final model presented in the manuscript corresponds to Adjusted Model 1.

Table S4. Evaluation of ambient ozone as a potential confounding variables to the final, linear mixed model. The final model included fixed effects for sex, ambient temperature, the presence of allergies and use of beta-agonists as well as random subject and day of study factors. A comparison of the percent change in FeNO per IQR is shown for the personal pollutant exposure metrics expressed per unit volume for the 0-lag period.

Table S5A. Effects of the oxidative burden exposure metrics expressed per unit mass and PM mass on the percent change in FeNO as modified by medication use (none, any, corticosteroids, beta-agonists, stimulants) for 0-, 1- and 2-day lag periods. Models were adjusted by the fixed effects (temperature, sex, the presence of allergies, eczema before the age of 2, occurrence of an asthma attack in the first year of life and use of beta-agonists) as well as random subject and day of study factors.

Table S5B. Effects of the oxidative burden exposure metrics per unit volume and PM mass on the percent change in FeNO as modified by medication use (none, any, corticosteroids, beta-agonists, stimulants) for 0-, 1- and 2-day lag periods. Models were adjusted by the fixed effects (temperature, sex, the presence of allergies, eczema before the age of 2, occurrence of an asthma attack in the first year of life and use of beta-agonists) as well as random subject and day of study factors.

Table S6A. Effects of oxidative burden exposure metrics expressed per unit mass and extracted PM mass from the personal exposure filter on the percent change in FeNO as modified by dust, mould, pollen and fur allergies for 0-, 1- and 2-day lag periods. Random and fixed effects included in mixed models are the same as Table S3A.

Table S6B. Effects of oxidative burden exposure metrics expressed per unit volume and PM mass concentration on the percent change in FeNO as modified by dust, mould, pollen and fur

allergies for 0-, 1- and 2-day lag periods. Random and fixed effects included in mixed models are the same as Table S3A.

Figure S1. Ambient ozone concentrations in Montreal, Canada. Mean daily concentrations and the associated standard deviations across four residential Environmental Canada-operated monitoring stations in Montreal are presented for the study period, October 6, 2009 to April 28, 2010.

Figure S2. Ascorbate and glutathione-related oxidative burden per micromole of individual quinone and metal solutions. All quinones and metal salts were diluted to a final concentration of 1.1 or 2.5 μM in high pressure liquid chromatography grade water containing 5% methanol for oxidative burden assessment.

Table S1. Comparison of select characteristics of the 70 children in the full data set and the 62 children included in the oxidative burden data set.

	**Full Data Set**<br>70 Children<br>N=675	**Oxidative Burden Data Set**<br>62 Children<br>N=249
Demographics		
Age in years, median (*IQR*)	10.0 (2.0)	10.0 (2.0)
Gender, n(*%*)		
Boys	49 (70)	43 (69)
Girls	21 (30)	19 (31)
Health status		
Allergies, n(*%*)	49 (70)	44 (71)
Eczema before age 2, n(*%*)	21 (31)*	17 (27)
Asthma attack in previous 12 months, n(*%*)	32 (46)	31 (50)
Parental asthma, n(*%*)	40 (57)	36 (58)
Medication use during monitoring		
Corticosteroids, n(*%*)	34 (49)	27 (44)
Stimulants, n(*%*)	10 (14)	6 (10)
Rescue medication (short acting beta agonist), n(*%*)	25 (36)	18 (29)
Personal Monitoring		
Temperature in ºC, median (*IQR*)	21.0 (1.6)	21.1 (2.0)
Relative Humidity in %, median (*IQR*)	40.4 (15.0)	43.1 (22.7)
$PM_{2.5}$ Mass Concentration in µg m^{-3}, median (*IQR*)	7.0 (9.7)	14.1 (10.8)
FeNO in ppb, median (*IQR*)	17.7 (25.2)	16.3 (24.8)

* Missing information from 2 children

Table S2A. Percent change in FeNO per IQR for pollutant exposure metrics expressed per unit mass for a 0-, 1- and 2-day lag periods with the addition of potential confounding variables to the base, linear mixed model. The base model included fixed effects for sex and ambient temperature as well as random subject and day of study factors. The final model included additional personal variables: the presence of allergies and use of beta-agonists.

		Glutathione (Percent Depletion per μg)			Ascorbate (Percent Depletion per μg)			Extracted Filter Mass (μg)		
		% Change per increase in IQR (95%CI)	% Change from Base Model	AIC	% Change per increase in IQR (95%CI)	% Change from Base Model	AIC	% Change per increase in IQR (95%CI)	% Change from Base Model	AIC
**0-day lag**	**Base Model**	6.3 (0.4,12.5)	-	232	1.2 (-3.5,6.0)	-	225	-0.68 (-4.2,3)	-	231
	Base Model+ **Allergies**	6.4 (0.5,12.6)	1.5%	232	1.3 (-3.4,6.2)	9.8%	224	-0.68 (-4.2,3)	0.0%	231
	Base Model+ **Asthma Attack in Past Year**	6.2 (0.3,12.4)	-2.3%	231	3.8 (-2.0,10)	223.5%	231	-0.73 (-4.3,3)	6.9%	231
	Base Model+ **Parental Asthma**	6.3 (0.4,12.5)	0.1%	233	1.2 (-3.5,6.1)	2.3%	226	-0.71 (-4.3,3)	3.4%	232
	Base Model+ **Eczema Before Age 2**	6.5 (0.6,12.7)	2.6%	235	3.6 (-2.2,9.8)	208.2%	230	-0.71 (-4.3,3)	3.4%	230
	Base Model+ **Beta-agonist Use**	6.1 (0.2,12.3)	-3.5%	231	1.2 (-3.5,6.1)	0.0%	229	-0.68 (-4.2,3)	0.0%	235
	Base Model+ **Corticosteroid Use**	6.3 (0.4,12.5)	-0.5%	234	1.2 (-3.5,6.0)	-0.8%	226	-0.66 (-4.2,3)	-3.4%	232
	**Final Model**	**6.6 (0.7,12.8)**	**4.4%**	**235**	**1.3 (-3.4,6.2)**	**9.9%**	**228**	**-0.68 (-4.3,3)**	**0.0%**	**234**
**1-day lag**	**Base Model**	6.2 (-1.0,14.0)	-	271	3.7 (-2.1,9.9)	-	231	0.23 (-4.2,4.9)	-	209
	Base Model+ **Allergies**	6.4 (-0.8,14.2)	3.1%	271	3.9 (-1.9,10.1)	4.7%	230	0.34 (-4.1,5.0)	45.5%	208
	Base Model+ **Asthma Attack in Past Year**	6.1 (-1.1,13.9)	-1.8%	271	3.8 (-2.0,10)	1.9%	231	0.20 (-4.2,4.8)	-14.8%	210
	Base Model+ **Parental Asthma**	6.3 (-1.0.0,14)	0.4%	272	3.7 (-2.1,9.9)	-0.7%	232	0.23 (-4.2,4.9)	-2.3%	210
	Base Model+ **Eczema Before Age 2**	5.9 (-1.2,13.6)	-4.9%	273	3.6 (-2.2,9.8)	-3.0%	230	0.21 (-4.2,4.9)	-8.0%	209
	Base Model+ **Beta-agonist Use**	5.9 (-1.3,13.6)	-5.5%	269	3.8 (-2.0,10)	2.2%	234	0.08 (-4.3,4.7)	-67.1%	212
	Base Model+ **Corticosteroid Use**	6.2 (-1.1,13.9)	-0.9%	272	3.6 (-2.2,9.8)	-2.3%	232	0.28 (-4.2,4.9)	20.5%	211
	**Final Model**	**6.1 (-1.1,13.8)**	**-1.7%**	**273**	**4 (-1.9,10.2)**	**6.8%**	**234**	**0.18 (-4.2,4.8)**	**-20.5%**	**211**
**2-day lag**	**Base Model**	11.2 (3.9,18.9)	-	240	2.0 (-3.4,7.7)	-	210	2.5 (-1.7,6.8)	-	197
	Base Model+ **Allergies**	11.3 (4.0,19.0)	1.0%	240	2.2 (-3.2,7.9)	7.6%	210	2.5 (-1.6,6.9)	3.0%	196
	Base Model+ **Asthma Attack in Past Year**	11.0 (3.8,18.8)	-1.0%	240	2.1 (-3.3,7.8)	5.1%	211	2.4 (-1.7,6.7)	-2.2%	197
	Base Model+ **Parental Asthma**	11.2 (3.9,19.0)	0.5%	241	2.0 (-3.4,7.7)	2.1%	211	2.4 (-1.7,6.8)	-0.6%	198
	Base Model+ **Eczema Before Age 2**	10.9 (3.6,18.6)	-2.5%	239	1.9 (-3.5,7.6)	-5.9%	209	2.4 (-1.7,6.7)	-2.8%	195
	Base Model+ **Beta-agonist Use**	10.8 (3.6,18.6)	-2.9%	243	2.1 (-3.3,7.8)	2.9%	214	2.0 (-2.0,6.2)	-18.4%	197
	Base Model+ **Corticosteroid Use**	11.1 (3.9,18.9)	-0.3%	242	2.0 (-3.4,7.7)	-1.6%	212	2.5 (-1.7,6.8)	1.4%	198
	**Final Model**	**11.0 (3.7,18.7)**	**-1.8%**	**242**	**2.2 (-3.2,7.9)**	**10.3%**	**213**	**2.1 (-2.0,6.3)**	**-15.3%**	**196**

Table S2B. Percent change in FeNO per IQR for pollutant exposure metrics expressed per unit volume for a 0-, 1- and 2-day lag periods with the addition of potential confounding variables to the base, linear mixed model. The base model included fixed effects for sex and ambient temperature as well as random subject and day of study factors. The final model included additional personal variables: the presence of allergies and use of beta-agonists.

		Glutathione (Percent Depletion per m^3)			Ascorbate (Percent Depletion per m^3)			PM Mass Concentration ($\mu g\ m^{-3}$)		
		**% Change per increase in IQR (95%CI)**	**% Change from Base Model**	**AIC**	**% Change per increase in IQR (95%CI)**	**% Change from Base Model**	**AIC**	**% Change per increase in IQR (95%CI)**	**% Change from Base Model**	**AIC**
**0-day lag**	**Base Model**	5.7 (0.1,11.7)	-	229	0.9 (-4.1,6.2)	-	222	-0.34 (-2.8,2.2)	-	230
	Base Model+ **Allergies**	5.8 (0.2,11.8)	1.5%	229	1.0 (-4.0,6.4)	13.7%	221	-0.33 (-2.8,2.2)	-3.1%	230
	Base Model+ **Asthma Attack in Past Year**	5.6 (0.0,11.6)	-2.3%	228	0.9 (-4.2,6.2)	-2.8%	222	-0.34 (-2.8,2.2)	0.0%	230
	Base Model+ **Parental Asthma**	5.7 (0.1,11.7)	0.0%	230	0.9 (-4.1,6.3)	2.7%	223	-0.34 (-2.8,2.2)	0.0%	231
	Base Model+ **Eczema Before Age 2**	5.5 (-0.1,11.5)	-3.5%	228	0.8 (-4.2,6.1)	-12.3%	221	-0.37 (-2.8,2.1)	6.2%	229
	Base Model+ **Beta-agonist Use**	5.9 (0.2,11.9)	2.7%	233	0.9 (-4.2,6.3)	0.1%	225	-0.35 (-2.8,2.2)	3.1%	234
	Base Model+ **Corticosteroid Use**	5.7 (0,11.7.0)	-0.6%	231	0.9 (-4.1,6.2)	-1.3%	223	-0.34 (-2.8,2.2)	0.0%	232
	**Final Model**	**6.0 (0.3,12.0)**	**4.5%**	**232**	**1.1 (-4.0,6.4)**	**14.0%**	**225**	**-0.34 (-2.8,2.2)**	**0.0%**	**234**
**1-day lag**	**Base Model**	6.4 (-0.5,13.8)	-	267	4.2 (-2.0,10.8)	-	227	-3.0 (-5.7,-0.2)	-	204
	Base Model+ **Allergies**	6.6 (-0.3,13.9)	2.6%	267	4.4 (-1.8,11.1)	4.4%	227	-3.0 (-5.7,-0.2)	-0.4%	203
	Base Model+ **Asthma Attack in Past Year**	6.3 (-0.6,13.7)	-1.5%	267	4.3 (-1.9,10.9)	1.9%	228	-3.0 (-5.7,-0.2)	0.0%	230
	Base Model+ **Parental Asthma**	6.4 (-0.5,13.8)	0.3%	268	4.2 (-2.0,10.8)	-0.6%	229	-3.0 (-5.7,-0.2)	0.0%	205
	Base Model+ **Eczema Before Age 2**	6.1 (-0.7,13.4)	-4.6%	270	4.1 (-2.1,10.7)	-2.5%	227	-3.0 (-5.7,-0.2)	0.7%	229
	Base Model+ **Beta-agonist Use**	6.1 (-0.8,13.4)	-4.7%	266	4.3 (-1.9,10.9)	1.8%	231	-3.1 (-5.8,-0.4)	5.1%	206
	Base Model+ **Corticosteroid Use**	6.3 (-0.5,13.7)	-0.9%	269	4.1 (-2.1,10.7)	-2.3%	229	-3.0 (-5.7,-0.2)	0.0%	206
	**Final Model**	**6.3 (-0.6,13.6)**	**-1.9%**	**269**	**4.5 (-1.8,11.1)**	**6.1%**	**230**	**-3.1 (-5.8,-0.4)**	**4.7%**	**205**
**2-day lag**	**Base Model**	10.7 (3.6,18.2)	-	237	1.8 (-4.0,8.0)	-	207	-0.56 (-3.2,2.1)	-	197
	Base Model+ **Allergies**	10.8 (3.7,18.3)	0.9%	237	2.0 (-3.9,8.2)	9.5%	206	-0.53 (-3.1,2.1)	-5.5%	196
	Base Model+ **Asthma Attack in Past Year**	10.6 (3.5,18.1)	-0.9%	237	1.9 (-3.9,8.1)	6.2%	208	-0.56 (-3.2,2.1)	0.0%	197
	Base Model+ **Parental Asthma**	10.7 (3.6,18.3)	0.5%	238	1.8 (-4.0,8.0)	2.3%	208	-0.56 (-3.2,2.1)	0.0%	198
	Base Model+ **Eczema Before Age 2**	10.4 (3.3,17.9)	-2.3%	236	1.7 (-4.1,7.8)	-6.0%	206	-0.59 (-3.2,2.1)	5.5%	195
	Base Model+ **Beta-agonist Use**	10.3 (3.3,17.8)	-3.1%	240	1.8 (-4.0,8.0)	2.4%	211	-0.52 (-3.1,2.1)	-7.4%	197
	Base Model+ **Corticosteroid Use**	10.6 (3.5,18.2)	-0.3%	239	1.8 (-4.1,7.9)	-2.2%	209	-0.57 (-3.2,2.1)	1.8%	199
	**Final Model**	**10.4 (3.4,18.0)**	**-2.1%**	**239**	**2.0 (-3.8,8.2)**	**11.7%**	**210**	**-0.5 (-3.0,2.1)**	**-11.1%**	**197**

Table S3A. Comparison of the percent change in FeNO per IQR for pollutant exposure metrics expressed per unit mass for a 0-, 1- and 2-day lag periods in model using varying levels of model adjustment. The base model included fixed effects for sex and ambient temperature as well as random subject and day of study factors. Additional personal variables were included adjusted models. The final model presented in the manuscript corresponds to Adjusted Model 1.

		**Co-Variates**						**% Change per Increase in IQR**		
		**Sex**	**Ambient temp**	**Use of beta agonist**	**Presence of allergies**	**Eczema before age 2**	**Asthma attack last 12 months**	**0-day lag**	**1-day lag**	**2-day lag**
**Glutathione Depletion μg^{-1}**	Base Model	x	x					6.3 (0.4,12.5)	6.2 (-1,14)	11.2 (3.9,18.9)
	Final Model	x	x	x	x			6.6 (0.7,12.8)	6.1 (-1.1,13.8)	11.0 (3.7,18.7)
	Over Adjusted Model	x	x	x	x	x	x	6.2 (0.2,12.4)	5.6 (-1.5,13.3)	10.5 (3.3,18.2)
**Ascorbate Depletion μg^{-1}**	Base Model	x	x					1.2 (-3.5,6.0)	3.7 (-2.1,9.9)	2.0 (-3.4,7.7)
	Final Model	x	x	x	x			1.3 (-3.4,6.2)	4.0 (-1.9,10.2)	2.2 (-3.2,7.9)
	Over Adjusted Model	x	x	x	x	x	x	1.2 (-3.5,6.1)	3.9 (-1.9,10.1)	2.2 (-3.2,7.9)
**Extracted Filter Mass (µg)**	Base Model	x	x					-0.68 (-4.2,3)	0.23 (-4.2,4.9)	2.5 (-1.7,6.8)
	Final Model	x	x	x	x			-0.68 (-4.3,3)	0.18 (-4.2,4.8)	2.1 (-2.0,6.3)
	Over Adjusted Model	x	x	x	x	x	x	-0.73 (-4.3,3)	0.13 (-4.3,4.7)	1.9 (-2.1,6.1)

Table S3B. Comparison of the percent change in FeNO per IQR for pollutant exposure metrics expressed per unit volume for a 0-, 1- and 2-day lag periods in model using varying levels of model adjustment. The base model included fixed effects for sex and ambient temperature as well as random subject and day of study factors. Additional personal variables were included adjusted models. The final model presented in the manuscript corresponds to Adjusted Model 1.

		**Co-Variates**						**% Change per Increase in IQR**		
		**Sex**	**Ambient temp**	**Use of beta agonist**	**Presence of allergies**	**Eczema before age 2**	**Asthma attack last 12 months**	**0-day lag**	**1-day lag**	**2-day lag**
**Glutathione Depletion m^{-3}**	Base Model	x	x					5.7 (0.1,11.7)	6.4 (-0.5,13.8)	10.7 (3.6,18.2)
	Final Model	x	x	x	x			6.0 (0.3,12.0)	6.3 (-0.6,13.6)	10.4 (3.4,18.0)
	Over Adjusted Model	x	x	x	x	x	x	5.6 (-0.1,11.7)	5.8 (-1.0,13.1)	10.0 (3.0,17.5)
**Ascorbate Depletion m^{-3}**	Base Model	x	x					0.9 (-4.1,6.2)	4.2 (-2.0,10.8)	1.8 (-4.0,8.0)
	Final Model	x	x	x	x			1.1 (-4,6.4.0)	4.5 (-1.8,11.1)	2.0 (-3.8,8.2)
	Over Adjusted Model	x	x	x	x	x	x	0.9 (-4.2,6.3)	4.5 (-1.8,11.1)	2.0 (-3.8,8.2)
**PM Mass (μg m^{-3})**	Base Model	x	x					-0.34 (-2.8,2.2)	-3.0 (-5.7,-0.2)	-0.56 (-3.2,2.1)
	Final Model	x	x	x	x			-0.34 (-2.8,2.2)	-3.1 (-5.8,-0.4)	-0.50 (-3.0,2.1)
	Over Adjusted Model	x	x	x	x	x	x	-0.39 (-2.8,2.1)	-3.2 (-5.9,-0.4)	-0.52 (-3.1,2.1)

Table S4. Evaluation of ambient ozone as a potential confounding variables to the final, linear mixed model. The final model included fixed effects for sex, ambient temperature, the presence of allergies and use of beta-agonists as well as random subject and day of study factors. A comparison of the percent change in FeNO per IQR is shown for the personal pollutant exposure metrics expressed per unit volume for the 0-lag period.

		**% Change per increase across the IQR for the 0-day lag period**
**Glutathione Depletion m^{-3}**	Final Model	6.0 (0.3,12.0)
	Final Model + Ambient Ozone	5.8 (0.1,11.8)
**Ascorbate Depletion m^{-3}**	Final Model	1.1 (-4,6.4.0)
	Final Model + Ambient Ozone	1.0 (-4.1,6.3)
**PM Mass ($\mu g\ m^{-3}$)**	Final Model	-0.34 (-2.8,2.2)
	Final Model + Ambient Ozone	-0.45 (-2.9,2.0)

Table S5A. Effects of the oxidative burden exposure metrics expressed per unit mass and PM mass on the percent change in FeNO as modified by medication use (none, any, corticosteroids, beta-agonists, stimulants) for 0-, 1- and 2-day lag periods. Models were adjusted by the fixed effects (temperature, sex, the presence of allergies, eczema before the age of 2, occurrence of an asthma attack in the first year of life and use of beta-agonists) as well as random subject and day of study factors.

Exposure Lag Period		Glutathione (Percent Depletion per µg)			Ascorbate (Percent Depletion per µg)			Extracted Filter Mass (µg)		
		% Change per increase in IQR (95% CI)	Total N	Interaction Term	% Change per increase in IQR (95% CI)	Total N	Interaction Term	% Change per increase in IQR (95% CI)	Total N	Interaction Term
**0-day lag**	**All Children**	6.6, (0.7, 12.8)	208		1.3, (-3.4, 6.2)	212		-0.7, (-4.3, 3)	214	
	**No Medications**	7.4, (-2.0, 17.6)	91	p=0.71	-3.3, (-10.4, 4.3)	94	p=0.10	1.6, (-3.2, 6.6)	93	p=0.25
	**Any Medications**	5.4, (-1.4, 12.6)	117		4.9, (-1.3, 11.5)	118		-2.4, (-7.2, 2.7)	121	
	**No Corticosteroids**	6.6, (-3.2, 17.3)	98	p=0.97	-3.3, (-10.2, 4.1)	100	p=0.18	1.8, (-2.9, 6.7)	100	p=0.20
	**Irregular Corticosteroids**	5.6, (-0.9, 12.5)	110		5.1, (-1.3, 11.8)	112		-2.6, (-7.4, 2.5)	114	
	**Regular Corticosteroids**	4.7, (-4.7, 15)	48		False Convergence			False Convergence		
	**No Beta-agonists**	False Convergence		p=0.60	-1.0, (-6.4, 4.6)	179	p=0.60	0.3, (-4.7, 5.5)	180	p=0.89
	**Beta-agonists**	8.1, (-5.6, 23.8)	34		6.6, (-8.8, 24.5)	33		-1.0, (-7.7, 6.3)	34	
**1-day lag**	**All Children**	6.1, (-1.1, 13.8)	191		4, (-1.9, 10.2)	193		0.2, (-4.2, 4.8)	192	
	**No Medications**	18, (5.4, 32.2)	84	p=0.07	11.6, (0.2, 24.3)	85	p=0.21	-1.5, (-6.8, 4.0)	83	p=0.43
	**Any Medications**	0.9, (-7.4, 9.9)	107		-0.1, (-7.1, 7.4)	108		1.5, (-4.4, 7.9)	109	
	**No Corticosteroids**	17.4, (4.9, 31.4)	91	p=0.13	10.7, (0.8, 21.6)	92	p=0.31	-1.0, (-6.2, 4.5)	90	p=0.68
	**Irregular Corticosteroids**	0.3, (-7.9, 9.2)	100		-0.7, (-7.6, 6.6)	101		0.9, (-5.4, 7.7)	102	
	**Regular Corticosteroids**	False Convergence			2.7, (-6.9, 13.3)	43		5.8, (-3.4, 16.0)	44	
	**No Beta-agonists**	5.7, (-2.4, 14.4)	160	p=0.76	5.9, (-0.7, 12.8)	162	p=0.13	1.6, (-3.7, 7.2)	161	p=0.40
	**Beta-agonists**	7.1, (-3.2, 18.4)	31		8.7, (-7.3, 27.4)	31		0.0, (-7.1, 7.6)	31	
**2-day lag**	**All Children**	11, (3.7, 18.7)	172		2.2, (-3.2, 7.9)	173		2.1, (-2, 6.3)	173	
	**No Medications**	15.4, (4.8, 27.1)	76	p =0.17	7.5, (-2.5, 18.7)	75	p=0.25	1.8, (-3.1, 6.9)	73	p=0.97
	**Any Medications**	5.9, (-3.3, 16)	96		-1.9, (-8.1, 4.7)	98		2.1, (-4.5, 9.1)	100	
	**No Corticosteroids**	17.4, (6.2, 29.7)	82	p=0.10	8.0, (-1.4, 18.4)	81	p=0.12	1.9, (-2.3, 6.3)	80	p=0.18
	**Irregular Corticosteroids**	4.3, (-4.3, 13.6)	90		-2.3, (-8.6, 4.5)	92		1.5, (-4.8, 8.3)	93	
	**Regular Corticosteroids**	11.4, (-3.0, 27.8)	41		4.2, (-5.8, 15.2)	41		-2.4, (-8.6, 4.3)	42	
	**No Beta-agonists**	11.3, (2.7, 20.6)	143	p=0.90	1.4, (-5.0, 8.2)	144	p=0.25	3.9, (-2.0, 10.2)	143	p=0.59
	**Beta-agonists**	6.4, (-9.2, 24.6)	29		-0.1, (-21.4, 27)	29		-0.6, (-8.0, 7.4)	30	

Table S5B. Effects of the oxidative burden exposure metrics per unit volume and PM mass on the percent change in FeNO as modified by medication use (none, any, corticosteroids, beta-agonists, stimulants) for 0-, 1- and 2-day lag periods. Models were adjusted by the fixed effects (temperature, sex, the presence of allergies, eczema before the age of 2, occurrence of an asthma attack in the first year of life and use of beta-agonists) as well as random subject and day of study factors.

Exposure Lag Period		Glutathione (Percent Depletion per m^3)			Ascorbate (Percent Depletion per m^3)			PM Mass Concentration ($\mu g\ m^{-3}$)		
		**% Change per increase in IQR (95% CI)**	**Total N**	**Interaction Term**	**% Change per increase in IQR (95% CI)**	**Total N**	**Interaction Term**	**% Change per increase in IQR (95% CI)**	**Total N**	**Interaction Term**
**0-day lag**	**All Children**	6.0, (0.3, 12.0)	**208**		1.1, (-4.0, 6.4)	212		-0.3, (-2.8, 2.2)	214	
	**No Medications**	6.6, (-2.1, 16.1)	91	p=0.74	-3.1, (-10.5, 4.8)	94	p=0.15	1.3, (-1.9, 4.7)	93	p=0.11
	**Any Medications**	5.0, (-1.8, 12.3)	117		4.5, (-2.1, 11.5)	118		-2.4, (-6.3, 1.6)	121	
	**No Corticosteroids**	6.0, (-3.1, 15.9)	98	p=1.00	-3.2, (-10.5, 4.7)	100	p=0.29	1.5, (-1.7, 4.9)	100	p=0.26
	**Irregular Corticosteroids**	5.3, (-1.3, 12.3)	110		4.6, (-2.2, 11.8)	112		-2.3, (-6.2, 1.7)	114	
	**Regular Corticosteroids**	4.4, (-3.8, 13.3)	48		False Convergence			False Convergence		
	**No Beta-agonists**	False Convergence		p=0.60	-1.2, (-6.6, 4.6)	179	p=0.60	-1.0, (-3.6, 1.6)	180	p=0.89
	**Beta-agonists**	8.1, (-5.5, 23.7)	34		6.0, (-8.5, 22.9)	33		-0.5, (-9.0, 8.8)	34	
**1-day lag**	**All Children**	6.3, (-0.6, 13.6)	191		4.5, (-1.8, 11.1)	193		-3.1, (-5.8, -0.4)	192	
	**No Medications**	17.4, (5.4, 30.8)	84	p=0.08	11.5, (0.6, 23.5)	85	p=0.20	-0.9, (-4.5, 2.8)	83	p=0.13
	**Any Medications**	1.0, (-7.4, 10.0)	107		0.0, (-7.6, 8.3)	108		-4.9, (-8.8, -0.7)	109	
	**No Corticosteroids**	16.3, (4.8, 29.1)	91	p=0.12	11.9, (1.2, 23.8)	92	p=0.28	-0.9, (-4.3, 2.6)	90	p=0.26
	**Irregular Corticosteroids**	0.4, (-7.6, 9.1)	100		-0.6, (-7.9, 7.2)	101		-5.0, (-9.1, -0.7)	102	
	**Regular Corticosteroids**	False Convergence			5.2, (-6.1, 17.9)	43		-4.4, (-8.8, 0.3)	44	
	**No Beta-agonists**	5.9, (-1.8, 14.2)	160	p=0.78	6.4, (-0.4, 13.7)	162	p=0.10	-3.6, (-6.1, -0.9)	161	p=0.07
	**Beta-agonists**	7.5, (-3.4, 19.5)	31		8.8, (-7.7, 28.2)	31		-3.6, (-8.7, 1.8)	31	
**2-day lag**	**All Children**	10.4, (3.4, 18)	172		2.0, (-3.8, 8.2)	173		-0.5, (-3.0, 2.1)	173	
	**No Medications**	14.7, (4.4, 26.1)	76	p =0.21	7.7, (-1.5, 17.8)	75	p=0.18	-0.9, (-3.6, 1.9)	73	p=0.73
	**Any Medications**	5.2, (-3.7, 14.8)	96		-2.8, (-9.4, 4.3)	98		0.0, (-4.0, 4.3)	100	
	**No Corticosteroids**	15.8, (5.5, 27.2)	82	p=0.11	7.8, (-0.9, 17.3)	81	p=0.08	-0.7, (-3.3, 2.0)	80	p=0.98
	**Irregular Corticosteroids**	3.8, (-4.8, 13.3)	90		-3.2, (-9.9, 3.9)	92		-0.1, (-4.4, 4.5)	93	
	**Regular Corticosteroids**	10.1, (-2.9, 24.8)	41		4.6, (-6.4, 16.8)	41		-0.9, (-4.6, 2.9)	42	
	**No Beta-agonists**	10.7, (2.4, 19.7)	143	p=0.82	1.4, (-5.1, 8.5)	144	p=0.26	-0.2, (-2.8, 2.5)	143	p=0.61
	**Beta-agonists**	6.2, (-9.3, 24.2)	29		0.6, (-20.6, 27.4)	29		-3.4, (-9.6, 3.3)	30	

Table S6A. Effects of oxidative burden exposure metrics expressed per unit mass and extracted PM mass from the personal exposure filter on the percent change in FeNO as modified by dust, mould, pollen and fur allergies for 0-, 1- and 2-day lag periods. Random and fixed effects included in mixed models are the same as Table S3A.

Exposure Lag Period		Glutathione (Percent Depletion per µg)			Ascorbate (Percent Depletion per µg)			Extracted Filter Mass (µg)		
		% Change per increase in IQR (95% CI)	Total N	Interaction Term	% Change per increase in IQR (95% CI)	Total N	Interaction Term	% Change per increase in IQR (95% CI)	Total N	Interaction Term
**0-day lag**	**All Children**	6.6, (0.7, 12.8)	**208**		1.3, (-3.4, 6.2)	212		-0.7 (-4.2,3.0)	214	
	**No Allergy**	10.3, (-3.4, 26)	61	p=0.43	2.7, (-8.3, 15)	62	p=0.72	False Convergence		p=0.22
	**Any Allergy**	4.6, (-1.8, 11.5)	141		-0.2, (-5.7, 5.6)	150		0.5, (-4.2, 5.4)	153	
	**No Dust Allergy**	8.8, (-1.8, 20.5)	94	p=0.47	1.8, (-5.8, 10.0)	97	p=0.73	-3.7, (-8.8, 1.7)	97	p=0.10
	**Dust Allergy**	4.2, (-2.5, 11.4)	114		0.1, (-6.1, 6.9)	115		1.2, (-4.1, 6.8)	117	
	**No Mould Allergy**	6.4, (0.0, 13.2)	168	p=0.72	2.1, (-3.3, 7.8)	172	p=0.59	-1.8, (-5.5, 2.2)	172	p=0.06
	**Mould Allergy**	10.2, (-5.4, 28.4)	40		-0.8, (-11.5, 11.2)	40		-1.9, (-6.1, 2.4)	42	
	**No Pollen Allergy**	5.7, (-1.4, 13.3)	127	p=0.75	1.8, (-4.7, 8.8)	131	p=0.91	-0.2, (-4.4, 4.1)	133	p=0.71
	**Pollen Allergy**	8.3, (-2.7, 20.4)	81		0.8, (-7.7, 10.1)	81		-3, (-11.8, 6.6)	81	
	**No Fur Allergy**	8.7, (1.0, 17.1)	125	p=0.38	2.8, (-5, 11.2)	127	p=0.70	-1.1, (-5, 2.9)	127	p=0.58
	**Fur Allergy**	2.1, (-6.3, 11.3)	83		0.1, (-6.5, 7.2)	85		-0.2, (-9.1, 9.6)	87	
**1 day lag**	**All Children**	6.1, (-1.1, 13.9)	191		4.0, (-1.9, 10.2)	193		0.2, (-4.2, 4.8)	192	
	**No Allergy**	3.4, (-10.1, 18.9)	57	p=0.74	3.0, (-8.2, 15.5)	60	p=0.36	2.1, (-5.4, 10.1)	58	p=0.97
	**Any Allergy**	6.9, (-1.3, 15.8)	134		3.8, (-3.4, 11.5)	133		-0.2, (-5.5, 5.3)	134	
	**No Dust Allergy**	6.2, (-5.3, 19.0)	87	p=0.72	2.2, (-6.1, 11.2)	91	p=0.57	0.4, (-6.0, 7.2)	90	p=0.73
	**Dust Allergy**	3.5, (-4.9, 12.7)	104		5.5, (-3.1, 14.9)	102		-0.7, (-6.8, 5.8)	102	
	**No Mould Allergy**	6.4, (-1.1, 14.5)	156	p=0.80	2.8, (-3.5, 9.6)	161	p=0.31	-0.3, (-4.9, 4.6)	159	p=0.59
	**Mould Allergy**	11.6, (-9.3, 37.2)	35		19.3, (-3.8, 48.0)	32		3.6, (-10.8, 20.3)	33	
	**No Pollen Allergy**	7.6, (-0.7, 16.7)	114	p=0.33	9.0, (0.7, 18.0)	118	p=0.12	-0.3, (-4.5, 4.0)	118	p=0.80
	**Pollen Allergy**	0.1, (-12.1, 14.0)	77		-4.7, (-13.7, 5.4)	75		1.7, (-9.0, 13.7)	74	
	**No Fur Allergy**	8.7, (1.0, 17.1)	125	p=0.97	2.8, (-5.0, 11.2)	127	p=0.57	-1.1, (-5.0, 2.9)	127	p=0.49
	**Fur Allergy**	5.2, (-7.2, 19.2)	76		3.0, (-6.4, 13.3)	75		4.1, (-6.0, 15.3)	75	
**2 day lag**	**All Children**	11.0, (3.7, 18.7)	172		2.2 (-3.2,7.9)	173		2.1 (-2.0,6.3)	173	
	**No Allergy**	10.9, (3.7, 18.6)	52	p=0.16	False Convergence		p= 0.68	False Convergence		p=0.62
	**Any Allergy**	11.8, (3.5, 20.8)	120		2.3, (-4.2, 9.3)	116		2.3, (-3.2, 8.2)	120	
	**No Dust Allergy**	10.8, (-2.2, 25.5)	79	p=0.90	1.6, (-6.5, 10.3)	81	p=0.81	4.7, (-0.8, 10.5)	81	p=0.06
	**Dust Allergy**	10.6, (3.2, 18.6)	93		1.1, (-6.5, 9.3)	92		-0.3, (-6.0, 5.8)	92	
	**No Mould Allergy**	6.4, (-1.1, 14.5)	156	p=0.18	2.8, (-3.5, 9.6)	161	p=0.53	-0.3, (-4.9, 4.6)	159	p=0.33
	**Mould Allergy**	20.5, (3.4, 40.6)	33		10.1, (-7.1, 30.4)	30		-1.1, (-11.1, 10.1)	31	
	**No Pollen Allergy**	10.8, (2.9, 19.4)	100	p=0.99	4.3, (-3.3, 12.4)	103	p=0.96	0.6, (-3.3, 4.6)	103	p=0.53
	**Pollen Allergy**	11.4, (-1.2, 25.7)	72		False Convergence			4.3, (-4.4, 13.7)	70	
	**No Fur Allergy**	8.9, (0.9, 17.6)	104	p=0.38	1.1 (-6.4,9.2)	105	p=0.30	0.8, (-2.7, 4.3)	104	p=0.72
	**Fur Allergy**	13.8, (1.9, 27.2)	68		False Convergence			4.5, (-5.5, 15.4)	69	

Table S6B. Effects of oxidative burden exposure metrics expressed per unit volume and PM mass concentration on the percent change in FeNO as modified by dust, mould, pollen and fur allergies for 0-, 1- and 2-day lag periods. Random and fixed effects included in mixed models are the same as Table S3A.

Exposure Lag Period		Glutathione (Percent Depletion per m^3)			Ascorbate (Percent Depletion per m^3)			PM Mass Concentration ($\mu g\ m^{-3}$)		
		% Change per increase in IQR (95% CI)	Total N	Interaction Term	% Change per increase in IQR (95% CI)	Total N	Interaction Term	% Change per increase in IQR (95% CI)	Total N	Interaction Term
**0-day lag**	**All Children**	6.0, (0.3, 12.0)	208		1.1, (-4.0, 6.4)	212		-0.7 (-4.2,3.0)	214	
	**No Allergy**	8.5, (-4.2, 22.8)	61	p=0.54	1.3, (-8.7, 12.5)	62	p=0.85	False Convergence		p=0.30
	**Any Allergy**	4.6, (-1.7, 11.4)	141		-0.1, (-5.9, 6.0)	150		0.5, (-4.2, 5.4)	153	
	**No Dust Allergy**	7.1, (-2.5, 17.8)	94	p=0.60	1.2, (-6.4, 9.5)	97	p=0.82	-3.7, (-8.8, 1.7)	97	p=0.30
	**Dust Allergy**	4.1, (-2.4, 11.0)	114		0.2, (-5.7, 6.5)	115		1.2, (-4.1, 6.8)	117	
	**No Mould Allergy**	5.7, (-0.4, 12.1)	168	p=0.73	1.7, (-3.9, 7.5)	172	p=0.66	-1.8, (-5.5, 2.2)	172	p=0.19
	**Mould Allergy**	11, (-6.7, 32.1)	40		-0.6, (-11.4, 11.6)	40		-1.9, (-6.1, 2.4)	42	
	**No Pollen Allergy**	5.2, (-1.6, 12.5)	127	p=0.62	1.3, (-5.8, 8.9)	131	p=0.98	-0.2, (-4.4, 4.1)	133	p=0.94
	**Pollen Allergy**	7.3, (-3.1, 18.8)	81		1.0, (-7.1, 9.9)	81		-3, (-11.8, 6.6)	81	
	**No Fur Allergy**	7.7, (0.3, 15.7)	125	p=0.46	1.9, (-6.3, 11.0)	127	p=0.89	-1.1, (-5.0, 2.9)	127	p=0.75
	**Fur Allergy**	2.3, (-5.8, 11.1)	83		0.6, (-6.0, 7.7)	85		-0.2, (-9.1, 9.6)	87	
**1 day lag**	**All Children**	6.3, (-0.6, 13.6)	191		4.5, (-1.8, 11.1)	193		0.2, (-4.2, 4.8)	192	
	**No Allergy**	3.3, (-9.5, 18)	57	p=0.67	2.6, (-8.3, 14.7)	60	p=0.37	2.1, (-5.4, 10.1)	58	p=0.43
	**Any Allergy**	7.2, (-0.7, 15.8)	134		4.3, (-3.0, 12.2)	133		-0.2, (-5.5, 5.3)	134	
	**No Dust Allergy**	6.0, (-4.6, 17.7)	87	p=0.76	2.1, (-6.6, 11.6)	91	p=0.51	0.4, (-6.0, 7.2)	90	p=0.10
	**Dust Allergy**	4.2, (-4.5, 13.7)	104		5.7, (-2.3, 14.4)	102		-0.7, (-6.8, 5.8)	102	
	**No Mould Allergy**	6.7, (-0.5, 14.3)	156	p=0.71	3.2, (-3.2, 10.0)	161	p=0.41	-0.3, (-4.9, 4.6)	159	p=0.98
	**Mould Allergy**	12.2, (-9.9, 39.6)	35		13.2, (-5.5, 35.6)	32		3.6, (-10.8, 20.3)	33	
	**No Pollen Allergy**	8.2, (-0.1, 17.1)	114	p=0.30	9.6, (0.9, 19.0)	118	p=0.11	-0.3, (-4.5, 4.0)	118	p=0.88
	**Pollen Allergy**	0.2, (-11.5, 13.5)	77		-4, (-12.9, 5.9)	75		1.7, (-9.0, 13.7)	74	
	**No Fur Allergy**	7.7, (0.3, 15.7)	125	p=0.98	1.9, (-6.3, 11.0)	127	p=0.63	-1.1, (-5.0, 2.9)	127	p=0.18
	**Fur Allergy**	5.4, (-6.4, 18.6)	76		3.3, (-5.6, 12.9)	75		4.1, (-6.0, 15.3)	75	
**2 day lag**	**All Children**	10.4, (3.4, 18)	172		2, (-3.8, 8.2)	173		2.1 (-2.0,6.3)	173	
	**No Allergy**	False Convergence		p=0.17	False Convergence		p= 0.74	False Convergence		p=0.80
	**Any Allergy**	11.7, (3.1, 21.0)	120		2.3, (-4.5, 9.5)	116		2.3, (-3.2, 8.2)	120	
	**No Dust Allergy**	9.4, (-3.1, 23.6)	79	p=0.96	0.9, (-7.4, 10.1)	81	p=0.72	4.7, (-0.8, 10.5)	81	p=0.40
	**Dust Allergy**	10.6, (3.2, 18.5)	93		1.3, (-6.3, 9.6)	92		-0.3, (-6.0, 5.8)	92	
	**No Mould Allergy**	6.7, (-0.5, 14.3)	156	p=0.18	3.2, (-3.2, 10.0)	161	p=0.39	-0.3, (-4.9, 4.6)	159	p=0.74
	**Mould Allergy**	22.1, (4.0, 43.4)	33		9.8, (-4.3, 26.0)	30		-1.1, (-11.1, 10.1)	31	
	**No Pollen Allergy**	10.5, (2.5, 19.1)	100	p=0.99	3.9, (-3.7, 12.1)	103	p=0.98	0.6, (-3.3, 4.6)	103	p=0.57
	**Pollen Allergy**	10.9, (-1.2, 24.5)	72		False Convergence			4.3, (-4.4, 13.7)	70	
	**No Fur Allergy**	8.7, (0.4, 17.7)	104	p=0.36	0.4, (-7.4, 9.0)	105	p=0.30	0.8, (-2.7, 4.3)	104	p=0.72
	**Fur Allergy**	13.1, (1.9, 25.5)	68		False Convergence			4.5, (-5.5, 15.4)	69	

Figure S1. Ambient ozone concentrations in Montreal, Canada. Mean daily concentrations and the associated standard deviations across four residential Environmental Canada-operated monitoring stations in Montreal are presented for the study period, October 6, 2009 to April 28, 2010.

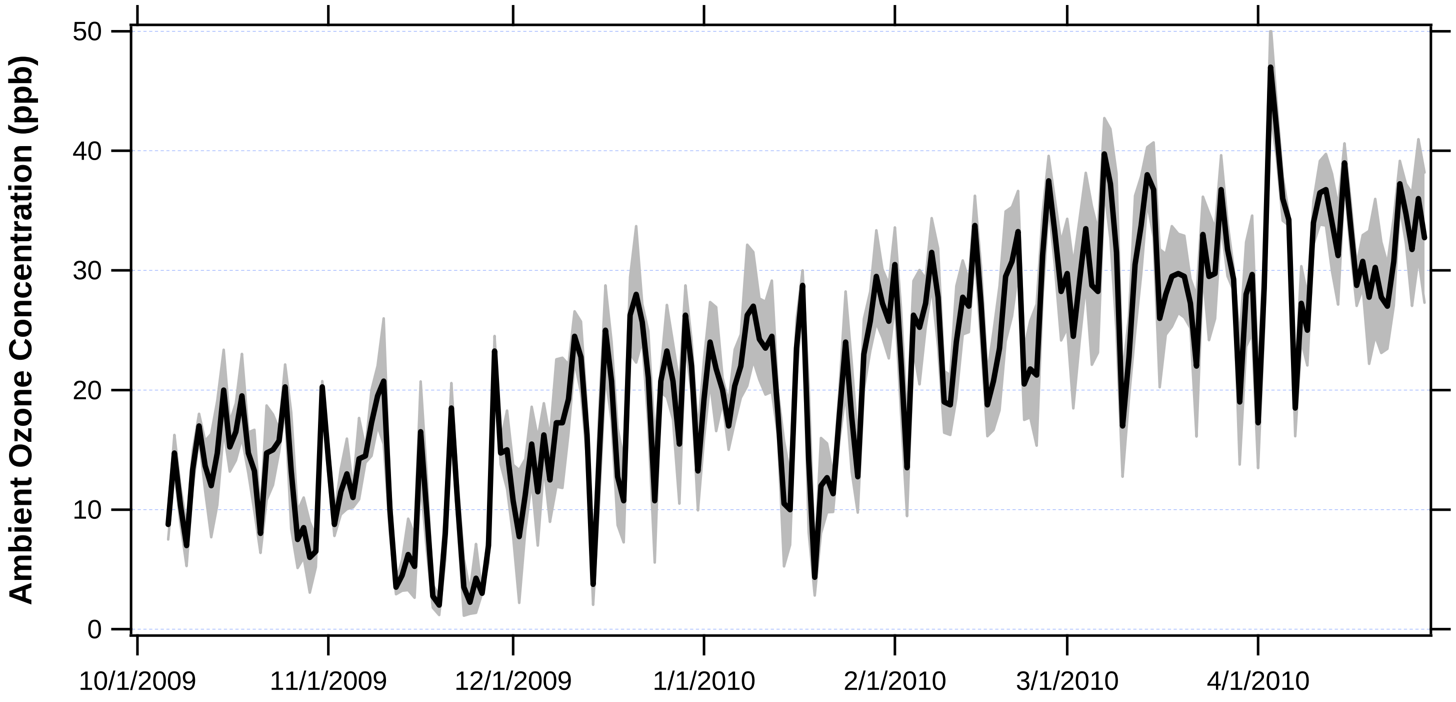

Figure S2. Ascorbate and glutathione-related oxidative burden per micromole of individual quinone and metal solutions. All quinones and metal salts were diluted to a final concentration of 1.1 or 2.5 µM in high pressure liquid chromatography grade water containing 5% methanol for oxidative burden assessment.

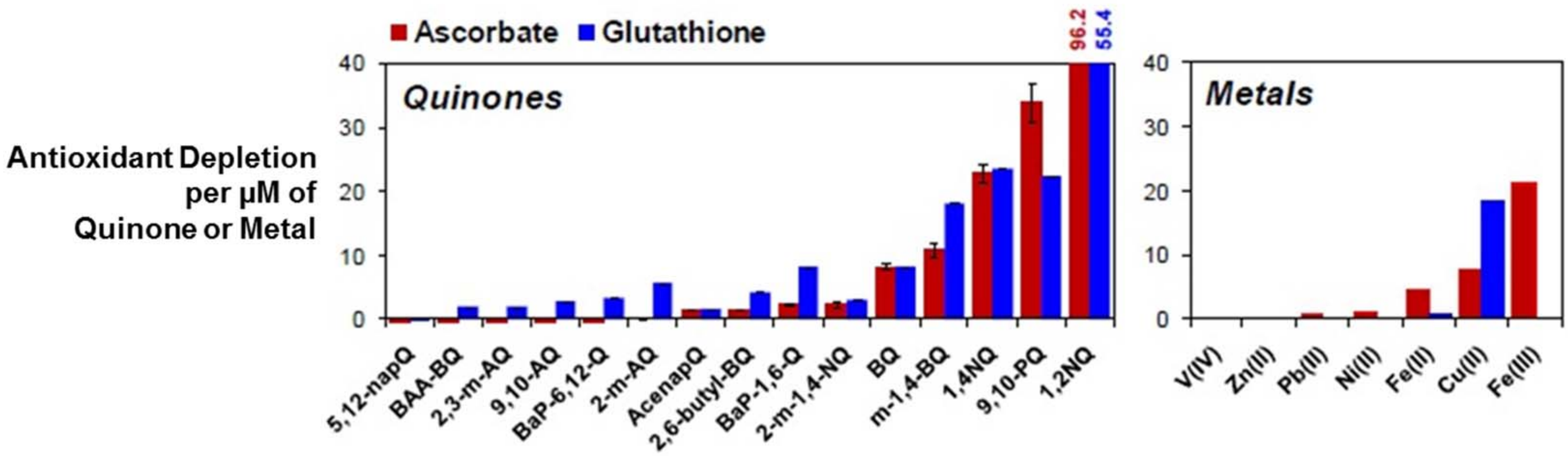

Abbreviations: Q - quinone; BQ – benzoquinone; AQ - anthraquinone; NQ - naphthoquinone.
